# Detecting modification of biomedical events using a deep parsing approach

**DOI:** 10.1186/1472-6947-12-S1-S4

**Published:** 2012-04-30

**Authors:** Andrew MacKinlay, David Martinez, Timothy Baldwin

**Affiliations:** 1 Department of Computing and Information Systems, University of Melbourne, VIC 3010, Australia; 2 NICTA Victoria Research Laboratories, University of Melbourne, VIC 3010, Australia

## Abstract

**Background:**

This work describes a system for identifying event mentions in bio-molecular research abstracts that are either speculative (e.g. *analysis of IkappaBalpha phosphorylation*, where it is not specified whether phosphorylation did or did not occur) or negated (e.g. *inhibition of IkappaBalpha phosphorylation*, where phosphorylation did *not *occur). The data comes from a standard dataset created for the BioNLP 2009 Shared Task. The system uses a machine-learning approach, where the features used for classification are a combination of shallow features derived from the words of the sentences and more complex features based on the semantic outputs produced by a deep parser.

**Method:**

To detect event modification, we use a Maximum Entropy learner with features extracted from the data relative to the trigger words of the events. The shallow features are bag-of-words features based on a small sliding context window of 3-4 tokens on either side of the trigger word. The deep parser features are derived from parses produced by the English Resource Grammar and the *RASP *parser. The outputs of these parsers are converted into the Minimal Recursion Semantics formalism, and from this, we extract features motivated by linguistics and the data itself. All of these features are combined to create training or test data for the machine learning algorithm.

**Results:**

Over the test data, our methods produce approximately a 4% absolute increase in F-score for detection of event modification compared to a baseline based only on the shallow bag-of-words features.

**Conclusions:**

Our results indicate that grammar-based techniques can enhance the accuracy of methods for detecting event modification.

## Introduction

This paper describes an automatic system for the recognition of bio-molecular events in biomedical literature. We base our research on the data from the BioNLP 2009 Shared Task [1], where events are defined relative to trigger words of different types, and the goal is to both identify the trigger words, and infer the role that each trigger word plays in a given event. As an illustration of the task, consider the input sentence:

(1) $$\begin{array}{l} {{[}_{protein}TRADD]}_{\text{1}}was\;the\;only\;protein\;that\;{{[}_{trigger}\mathrm{interacted}]}_{\text{4}}with\\ wild-type\;{{[}_{protein}TES2]}_{\text{2}}and\;not\;with\;isoleucine-mutated{{[}_{protein}TES2]}_{\text{3}}. \end{array}$$

where the words indicated in square brackets have been pre-identified as proteins as part of the Task specification. The event structure for this sentence, as defined in the shared task gold standard annotatations, is exemplified below:

(2) $$\begin{array}{l} \text{Event }evt_{\text{1}}\\ \text{TYPE }=\text{ BINDING}\\ \text{TRIGGER }=\;{{[}_{trigger}interacted]}_{\text{4}}\\ {\text{THEME}}_{\text{1}}=\;{{[}_{protein}TRADD]}_{\text{1}}\\ {\text{THEME}}_{\text{2}}=\;{{[}_{protein}TES2]}_{\text{2}} \end{array}$$

(3) $$\begin{array}{l} \text{Event }evt_{\text{2}}\\ \text{TYPE }=\text{ BINDING}\\ \text{TRIGGER }=\;{{[}_{trigger}interacted]}_{\text{4}}\\ {\text{THEME}}_{\text{1}}=\;{{[}_{protein}TRADD]}_{\text{1}}\\ {\text{THEME}}_{\text{2}}=\;{{[}_{protein}TES2]}_{\text{3}} \end{array}$$

(4) $$\begin{array}{l} {\text{Modification mod}}_{1}\\ \text{TYPE}=\text{N}\text{EGATION}\\ \text{THEME}={\text{evt}}_{2} \end{array}$$

The important points to note are: (a) events are defined as *n*-tuples via a unique trigger word and a number of arguments, indexed to words in the original text; (b) coordination (in the object NP in (1)) potentially leads to multiple event tuples; and (c) negated events are represented as nested structures, in the form of the base (unnegated) event and a meta-operator scoping over that event.

The shared consists of three component tasks. The first challenge (Task 1) identifies the trigger word for each event, together with its main arguments, e.g. (2) and (3) from above. Task 2 is devoted to the enrichment of events by identifying secondary arguments that further specify the event (e.g. LOCALISATION). Finally, Task 3 is focused on the identification of two types of event modification: NEGATION (such as (4)) and SPECULATION; SPECULATION modification indicates that the event was hedged or speculative (e.g. *analysis of IkappaBalpha phosphorylation*, where it is not clear whether *IkappaBalpha phosphorylation *occurred or not). Our primary interest in this paper is in Task 3, building on the outputs of Task 1.

There has been increasing attention in the detection of NEGATION and SPECULATION in scientific literature [2-4]. The importance of NEGATION, for example, can be illustrated via (1) above and a literature search task. Assume that the user were interested in identifying all biomedical papers which describe binding-style interaction between *TRADD *and *isoleucine-mutated TES2*. A classic text retrieval system would be able to identify that the abstract containing (1) refers to these two protein types, without being able to capture the nature of the interaction between them. Paired with a Task 1-style system, it would additionally be able to identify that this abstract specifically discusses binding-style interaction between the two proteins (and hence enhance retrieval precision). Only in combination with the predictions of a Task 3-style system, however, would it be able to additionally predict that this sentence describes the absence rather than presence of interaction, and hence not use this sentence as the basis of retrieving this abstract (further improving precision).

One of our principal interests is in the contribution of parsers to Task 3 performance. In essence, Task 3 involves determining the scope of NEGATION and SPECULATION operators over the event predicates predicted in Task 1. A parser which provides scoping information as first-order outputs seems, intuitively, to be an ideal solution to the task, and we seek to verify empirically that this intuition fits with the actuality of the task. As part of this exploration, we experiment with two parsers of varying linguistic precision and coverage (the English Resource Grammar and *RASP*), which we compare with a standalone bag-of-words baseline, and various hybrid techniques.

The contributions of this paper are as follows: (1) we experiment with a range of parsers, individually and in combination; (2) we compare our Task 3 systems to a bag-of-words baseline, in addition to hybridising bag-of-words and parsing features; and (3) we combine our Task 3 classifiers with a range of Task 1 systems, and systematically investigate the interaction between Task 1 and Task 3. In this, we achieve the best published results to date for NEGATION over the Task 3 test set, while for SPECULATION we achieved a score bettered by only one system in the original shared task.

### Related work

For the purposes of this paper, we treat Task 1 (trigger word detection) as a black box, and base our event modification classifiers on the output of a range of Task 1 systems from the original BioNLP 2009 Shared Task, namely: the best-performing Task 1 system of UTurku [5], the second-best Task 1 system of JULIELab [6], and the mid-ranking Task 1 system of NICTA [7]. For the majority of our experiments, we use the output of UTurku exclusively.

In the original shared task, only 6 systems participated in Task 3, of which 4 were based on hand-crafted rules operating over parser output, and developed based on the training data. The exceptions were the systems of [8] and [7]. The first system relied on decision trees trained over the BioScope corpus [4], which was specifically designed for the development of methods for detecting instances of event SPECULATION and NEGATION. The second system used a deep parser together with a machine learner, but did not combine parsers as we do and used a limited feature set.

The best performance for event modification in the original shared task was obtained by ConcordU [9], with a hand-coded grammar built on top of a syntactic parser. For SPECULATION they relied on active cognition verbs to define their syntactic patterns, while hand-picked clue words provided the rules for NEGATION detection.

The systems presented in [10-12] also relied on hand-crafted rules built by analysing the training data. [10] extended their ontology-driven pattern matching approach from Task 1, which suffered from low recall. [11] applied regular expressions to identify NEGATION trigger expressions, and defined rules based on deep parsing for both NEGATION and SPECULATION. [12] also built hand-crafted rules, and observed that most of the errors came from their Task 1 output, which provided less than 30% of the events necessary for full recall in Task 3.

One finding to come from the original shared task was the strong co-dependence between Task 1 and Task 3 results, i.e. that it is hard to perform well at Task 3 without a strong Task 1 system. We investigate this phenomenon relative to a selection of Task 1 systems in the 'Results and discussion' section.

Outside the shared task, other work has looked at tasks involving subsets of the modification types in Task 3. The *Negex *system [13] was developed as a general-purpose method for identifying phrasal NEGATION in medical texts, and relies on regular expressions over pre-identified trigger words, so is highly compatible with the BioNLP 2009 Shared Task data. The system was developed over clinical records, and for our experiments we used the version 2 implementation from http://code.google.com/p/negex. More recently, the CoNLL 2010 Shared Task [14] was concerned with detection of speculative language (or *hedging*), but not NEGATION, in biomedical text. The first subtask required identification of speculative sentences, and the second subtask required identification of the hedging cues and determination of their scope, with a set a biomedical articles as the primary focus. The second subtask in particular overlaps somewhat with Task 3 of the BioNLP 2009 Shared Task, where the participants were probably implicitly identifying cues and their scope (i.e. whether trigger words fell within it) to an extent. The task participants had some success in applying syntactic analyses to the problem - for example, [15] used dependency parses and deep LFG parses. Cue words were identified using a machine learning approach with mostly shallow features, and then a set of hand-crafted rules based on the syntactic analyses was applied to the cue words to identify their scope.

## Methods

Our basic approach is to parse the data, and construct feature vector inputs to a machine learner from the parser output(s). We build separate classifiers for each of the two subtasks of SPECULATION and NEGATION. In this section, we describe the parsers and feature extraction methodology.

### Deep parsing with the ERG

Intuitively, we would expect deep syntactico-semantic analysis to be useful in detecting both event NEGATION and SPECULATION, as knowledge of the relationships of possibly distant elements (such as the NEGATION particle *not*) to a particular target word can provide valuable information for classification. Indeed, as noted above, syntactic analysis of some kind was found to be useful for this task (e.g. [9]) and related tasks (e.g. [15])

Further to this, it was our intention to evaluate the utility of deep parsing [16] for the task, rather than a shallower annotation such as the output of a dependency parser. With this in mind, we selected the English Resource Grammar (*ERG*: [17,18]), an open-source, broad-coverage high-precision grammar of English in the HPSG framework [19]; the experiments reported in this paper are based on the '0902' version of the grammar. We combine the *ERG *with the *PET *parsing engine [20] in this work.

While the *ERG *is relatively robust across different domains, it is a general-purpose resource, and there are some aspects of the language used in the biomedical abstracts that cause difficulties; unknown word handling is especially important given the nature of terms in the domain. Fortunately we can make some optimisations to mitigate this. The GENIA tagger [21] provides both POS and named entity annotations, which we used to constrain the input to the *ERG *in two ways, using the *chart-mapping *machinery of [22]:

• Biological named entities identified by the GENIA tagger are 'flagged as such, and the parser does not attempt to decompose them.

• POS tags are appended to each input token to constrain the token to an appropriate category if it is absent from the ERG lexicon.

In addition to producing parse trees and full Attribute-Value Matrices, the *ERG *can also produce output in particular semantic formalisms: Minimal Recursion Semantics (MRS: [23]) and the closely-related Robust Minimal Recursion Semantics (RMRS: [24]). For our feature generation here we make use of the latter, due to its compatibility with shallower parsers such as *RASP*.

While the *ERG *has various grammar-internal mechanisms for increasing coverage (e.g. allowing subject-verb number mismatch), it does not have any facility to construct parse fragments in the instance that no spanning parse is found for a given input. This inevitably restricts the coverage of the grammar, and in the case of the BioNLP 2009 Shared Task data, the sentence-level coverage was found to be a respectable but still imperfect 76%. Clearly, a fallback strategy is required for the 24% of sentences the *ERG *is unable to parse. Some methods to achieve this are discussed in the next section.

### Extending parse coverage

One obvious approach to augment the *ERG *and gain full coverage over all inputs is to combine it with a more simplistic bag-of-words approach, and this is indeed something we investigate. However in line with our intuition and experimental evidence that syntactico-semantic features are useful for the task, we also investigated improving the coverage by adding an alternative parser.

One obvious choice would be any of the dependency parses provided by the organisers of the shared task. These parses have some advantages - broad coverage over the data and being tuned to the biomedical domain being obvious ones. However, we wished to leverage off our previous feature engineering work with deriving salient indicators from RMRSs, and hence opted to use *RASP *[25], a broad-coverage general-purpose statistical parser, applying the method of [26] to generate RMRSs.

In our setup, we did not allow fragment analyses from *RASP *due to the difficulty of converting them to RMRS outputs and doubts about their reliability. Similar to our approach with the *ERG*, we only used the top-ranked parse.

Under these conditions, we found that *RASP *was able to achieve similar coverage to the *ERG*, obtaining a parse for 76% of the sentences in the development data. However, by taking the union of the sets of parseable inputs from the two parsers, we increase our sentence-level coverage to 93% of the development set, making features derived from RMRSs a far more realistic prospect as a means of detecting event modification.

### Feature extraction from RMRSs

**Figure 1:**
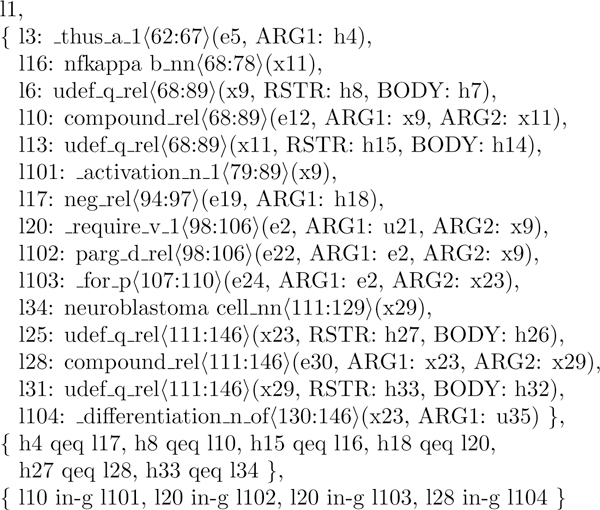
**A sample RMRS**. RMRS representation of the sentence *Thus NF-kappa B activation is not required for neuroblastoma cell differentiation *showing, in order, elementary predicates (each consisting of a label, predicate name, character span and arguments), qeq-constraints, and in-g constraints. The unlabelled first argument of each predicate is the mandatory *ARG0 *argument, which is closely linked to the predicate. The 'udef_q_rel' predicates are default quantifiers introduced to keep the RMRS well-formed, which do not have directly corresponding words in the sentence.

Figure 1 shows an RMRS obtained from one of the training documents. While there is insufficient space to give a complete treatment here, we highlight several aspects of importance to this paper. The primary component of an RMRS is bag of *elementary predicates*, or EPs. Each EP has:

1. A label, such as *l104*. The label indices are unique but arbitrarily assigned by the grammar. As such, they do not necessarily start at zero, and generally have increments of greater than one.

2. A predicate name, such as _*differentiation*_*n*_*1*. The *n *before the final digit indicates the word is a noun; a *v *denotes a verb, an *a *denotes an adjective or adverb, and a *q *denotes a quantifier, such as a determiner.

3. Character indices to the source sentence, such as 〈130:146〉, indicating the predicate corresponds to characters 130 to 146 in the source text.

4. A set of arguments.

Arguments can be variables such as *e30 *or *x23 *(where the first letter indicates the nature of the variable - *e *referring to events, *x *referring to entities and *u *indicating underspecified), or *handles *such as *h33*. The first argument is always *ARG0 *and is afforded special status, generally referring to the variable introduced by the predicate. Subsequent arguments are labelled according to the relation of the argument to the predicate. For open-class predicates such as verbs, these are non-committal names of the form *ARG*n, but follow certain conventions - for example, in English, the (deep) subject of the verb is generally *ARG1 *and the (deep) object is *ARG2*. Some closed-class words such as determiners and conjunctions follow different conventions for argument naming - this is visible for the *udef_q_rel *quantifiers in Figure 1. These handles are generally used in the *qeq constraints*, which relate a handle to a label, indicating a particular kind of outscoping relationship between the handle and the label - either that the handle and label are equal or that the handle is equal to the label apart from one or more quantifiers occuring between the two (the name is derived from 'equality modulo quantifiers'). Finally there are *in-g constraints *which indicate that labels can be treated as equal. For our purposes this simply affects which qeq constraints they participate in - for example from the in-g constraint *l28 in-g l104 *and the qeq constraint *h27 qeq l28*, we can also infer that *h27 qeq l104*.

In constructing features, we make use of:

• The *outscopes *relationship (specifically qeq-outscopes) - if EP *A *has a handle argument which qeq-outscopes the label of EP *B*, *A *is said to immediately outscope *B*; *outscopes *is the transitive closure of this. For example, in Figure 1, the EP *l3*: _*thus_a_1 *has a handle argument *h4 *as its *ARG1*, which in combination with the qeq constraint *h4 qeq l17 *means that *l3 *immediately outscopes the EP *l17*: *neg_rel*. Similarly *l17 *in turn immediately outscopes *l20*:_*require*_*v*_*1*. From the transitive closure, we can use both of these to infer that *l3 *also outscopes *l20*, since *l3 *outscopes something (in this case *l17*) which in turn outscopes *l20*.

• The *shared-argument *relationship, where EPs *C *and *D *refer to the same variable in one or more of their argument positions. For instance in Figure 1, *l28*: *compound_rel *shares its *ARG1 *with the *ARG0 *of *l104*: _*differentiation_n_of*, as both slots are filled by the same variable *x23*. We also in some cases make further restrictions on the types of arguments (*ARG0*, *RSTR*, etc) that may be shared on either end of the relationship.

### Feature sets and classification

Feature vectors for a given event are constructed on the basis of the trigger word for that event, which we assume has already been identified. We use the term *trigger EPs *to describe the EP(s) which correspond to that trigger word - i.e. those whose character span encompasses the trigger word. We have a potentially large set of related EPs (with the kinds of relationships described above), which we filter to create the various feature sets, as outlined below.

The following features are used to identify NEGATION. In each case, a general feature is set (e.g. NegOutscope2), as well as a specific one for the matching predicate.

• NegOutscope2: an EP in the RMRS belongs to a set of nine semantically negative predicates (e.g. _*unable_a *or _*never_a*) determined by manual examination of a small subset of the development data, and that EP outscopes a trigger EP.

• NegConjIndex: an EP in the RMRS belongs to a set of three negative conjunctions (_*nor_c, _not_c *and _*but+not_c*) identified from the grammar, and the negated daughter(s) of that EP are the *ARG0 *of a trigger EP.

• Arg0NegOutscopeeSA: an EP has an argument that matches the *ARG0 *of a trigger EP, which is in turn outscoped by the same set of negative EPs as for NegOutscope2. This feature is designed to catch trigger EPs which are nouns, where the dominating predicate is semantically negated.

• TrigPredProps: the predicate name of each trigger EP, as well as its POS.

The following are the features to identify SPECULATION. Once again, in each case, both a general feature and a special predicate-based feature are set.

• SpecVObj2+WN: one of a pre-identified seed set of six speculative verbs is found (e.g. _*test *or _*investigate*), where its *ARG2 *(i.e. object) is the *ARG0 *of a trigger EP. We additionally include WordNet sisters of the speculative verbs, and in the case that a WordNet sister matches, add an additional feature for the seed speculative verb (in the original set). The seed verbs were identified by examining a subset of the development data.

• ModalOutscope: a modal verb (e.g. *should*) outscopes a trigger EP.

• AnalysisSA: the *ARG0 *of the trigger EP is also an argument of an EP with the predicate name _*analysis*_*n*. Such constructions involving the word *analysis *are relatively frequent in speculative events in the data.

• ModAdj: any adjectival or adverbial EPs which have an *ARG1 *(corresponding to the modified noun or verb) which matches the *ARG0 *of the trigger EP (i.e. which are modifiers).

Both of these feature sets are the same as the most successful 'N4' and 'S3' feature sets used in [7].

### Combining RMRSs from different sources

Given that we have multiple potential sources of RMRSs to create feature vectors, there are several possible ways to combine them. The first is a fallback method. We have more confidence in the *ERG *parses and their ability to produce RMRSs for a number of reasons: the *ERG *is a deeper grammar, in contrast to the deliberate shallowness of the *RASP *parser, so we would expect, where it can find a parse, that its analyses would contain more useful information; additionally RMRS is closer to a native format for the *ERG*, as it is constructed compositionally as part of the parsing process, rather than in a post-processing step, as is the case with *RASP*. On the basis of this, we use the *ERG*-derived RMRS where it is available, and where it isn't, fall back to the RMRS derived from *RASP*.

An alternative is to place equal confidence in both sources of RMRSs. Each sentence will have zero, one, or two RMRSs available. In the first case where we have one RMRS, we construct features from it as usual. Where there are two RMRSs, we construct features from each, and take the union to form a single feature vector. A variant on this method is to produce the same merged output if there are multiple input RMRSs, but also produce a version of each feature that is tagged with the source of the RMRS. The intuition here is that while there are some commonalities between the RMRS outputs, each grammar may have different strengths and weaknesses in terms of producing RMRSs, so it may be useful for the machine learning algorithm to have (indirect) knowledge of which grammar produced the particular feature.

### Bag-of-words features

To evaluate the performance boost obtained through parsing relative to more naive methods, we also experimented with feature sets based on a bag-of-words approach with a sliding context window of tokens on either side of the token corresponding to the trigger, as determined by the tokenisation of the GENIA tagger, without crossing sentence boundaries. We evaluated a range of combinations of preceding and following context window sizes from 0 to 5 (never crossing sentence boundaries), and optimised the window size for each of the SPECULATION and NEGATION subtasks.

The bag-of-words context-window is robust and gives 100% coverage, so it gives us a chance at classifying the sentences which are not parseable using either parser. It is also possible that even on sentences we can parse with the *ERG *and/or *RASP*, the event modifications it can detect are at least partially complementary to those that are detectable with the RMRS-derived features, suggesting a combined approach.

### Classifier implementation

To produce training data to feed into a classifier, we parsed as many sentences as possible using the *ERG *and/or *RASP*, and used the output RMRSs to create training data, relying on the features described above. The construction of features, however, presupposes annotations for the events and trigger words. For producing training data, we used the provided trigger annotations. For the test phase, we simply use the outputs of the various Task 1 classifiers as a source of trigger annotations, selecting the combination with the best performance over the development set. We used a maximum entropy classifier, by applying Zhang Le's Maxent Toolkit (http://homepages.inf.ed.ac.uk/lzhang10/maxent_toolkit.html).

## Results and discussion

We test the impact of the two parsers - individually and in combination - on SPECULATION and NEGATION event modification over both the development data and over the test data provided for the shared task. The reason we use both datasets is that evaluation over the test data is possible only via a web form, with the restriction that only one run can be evaluated in each 24 hour time period, to maintain the sanctity of the test data. We thus carried out extensive experiments over the development data to fine-tune our feature engineering by applying combinations of different feature sets, including many not reported here. We apply only a representative set of classifiers to the test data. To explore the impact of the Task 1 results (trigger word detection) on event modification, we make use of the following Task 1 systems for the development and/or test datasets. Note that gold-standard annotations are not available for the test dataset, meaning that any experiments requiring gold-standard data can only be performed over the development dataset.

• The output of the UTurku system [5], which was the best-performing Task 1 system in the original shared task [DEV and TEST]

• The gold-standard annotations, to evaluate our methods in isolation of Task 1 classifier noise [DEV only]

• The outputs of the JULIELab and NICTA Task 1 classifiers, to explore the impact of Task 1 classifier performance on event modification [DEV only]

### Results over the development data

We present first the baseline bag-of-words results, and then our parser-based systems.

#### Bag-of-words baseline

We first carried out a series of experiments with different window sizes for the bag-of-words method over the development data, to determine the optimal window size for each of the NEGATION and SPECULATION subtasks. Using gold-standard Task 1 data and optimising over event modification F-score, we found that the optimal window size for SPECULATION was three words to either side of the event trigger word (signified as ${\text{W}}_{+3}^{-3}$), at an F-score of 48.3%. For NEGATION, the marginally wider window size of four words to the left and three words to the right (signified as ${\text{W}}_{+3}^{-4}$) produced the optimal F-score of 53.3% over the development data (once again based on gold-standard Task 1 annotations). Perhaps the most surprising thing about this relatively uninformed baseline is how well it can perform. These window size settings are used exclusively in the bag-of-words experiments presented in the remainder of this paper for the respective subtasks.

#### RMRSs and parser combination

In Table 1 we present the results over the development data using the UTurku classifier and gold-standard Task 1 annotations. We additionally include results for the rule-based *Negex *system [13] described earlier in the paper, as a benchmark for the NEGATION subtask. Recall that both the *ERG *and *RASP *have imperfect coverage over the data, meaning that in cases where bag-of-words features are not employed, the feature vector will consist of all negative features, and the classifier will fall back on the class priors to classify the instance in question.

**Table 1:** Results over development set

Mod	RMRS from	Extra	Gold			UTurku		
			R	P	F	R	P	F
SPECULATION	-	${\text{W}}_{+3}^{-3}$	42.9	55.4	48.3	19.0	33.3	24.2
SPECULATION	*RASP*	-	16.7	66.7	26.7	5.5	26.1	9.0
SPECULATION	*ERG*	-	20.2	68.0	31.2	10.7	56.2	18.0
SPECULATION	fb(*ERG*,*RASP*)	-	25.0	61.8	35.6	13.1	50.0	20.8
SPECULATION	cb(*ERG*,*RASP*)	-	15.5	59.1	24.5	10.7	52.9	17.8
SPECULATION	*ERG*	${\text{W}}_{+3}^{-3}$	45.2	59.4	51.3	20.2	34.0	**25.4**
SPECULATION	fb(*ERG*,*RASP*)	${\text{W}}_{+3}^{-3}$	45.2	60.3	**51.7**	16.7	31.1	21.7
SPECULATION	cb(*ERG*,*RASP*)	${\text{W}}_{+3}^{-3}$	40.5	54.8	46.6	19.0	32.0	23.9

NEGATION	-	*Negex*	32.7	32.7	32.7	22.6	17.8	19.9
NEGATION	-	${\text{W}}_{+3}^{-4}$	51.8	54.8	53.3	25.4	33.7	**29.0**
NEGATION	*RASP*	-	12.7	35.9	18.8	5.4	26.1	9.0
NEGATION	*ERG*	-	26.4	72.5	38.7	15.4	34.0	21.2
NEGATION	fb(*ERG*,*RASP*)	-	35.4	66.1	46.1	17.3	34.6	23.0
NEGATION	cb(*ERG*,*RASP*)	-	29.1	64.0	40.0	13.6	34.1	19.5
NEGATION	*ERG*	${\text{W}}_{+3}^{-4}$	45.4	48.5	47.0	18.2	26.3	21.5
NEGATION	fb(*ERG*,*RASP*)	${\text{W}}_{+3}^{-4}$	44.6	66.2	53.3	19.1	33.3	24.3
NEGATION	cb(*ERG*,*RASP*)	${\text{W}}_{+3}^{-4}$	50.9	59.0	**54.6**	21.8	32.0	26.0

Results over the development data using gold-standard Task 1 annotations and the UTurku Task 1 system ("fb" = fallback strategy, where we use the first source if possible, otherwise the second; "cb" = use undifferentiated RMRSs from each source to create feature vectors).

Firstly, for the pure RMRS-based features, there are obvious differences between the methods of RMRS construction. The standalone *ERG *produces respectable performance in NEGATION and acceptable performance in SPECULATION in relation to the baselines. In line with our predictions, the standalone *ERG *produces superior performance to the standalone *RASP*.

In terms of strategies for combining the features from different RMRSs, it seems that the fallback strategy (fb) is most effective: creating an RMRS from the *ERG *where possible, and otherwise from *RASP *produces a substantial performance boost over the standalone *ERG *strategy, which is consistent across SPECULATION and NEGATION, and both the gold-standard and UTurku outputs. This is interesting as for only 17% of the sentences in the data was there a *RASP *parse and not an *ERG *parse. It seems that there is relatively good compatibility between the features produced from these different RMRSs, so that features learnt from *RASP*-derived RMRSs can be used for *ERG*-derived RMRS output and vice versa. The strategy which combines every possible parse obtained from the *ERG *and *RASP *(cb) is generally less effective, with the one exception of NEGATION, where bag-of-words features are combined with the RMRS features. In fact, in the majority of cases, cb without bag-of-word features is inferior to using the *ERG *as a standalone parser.

When we combine the bag-of-words features with the RMRS-derived features, the results always improve over the equivalent RMRS results without bag-of-words, with recall being the primary benefactor. The cb strategy appears to benefit most from the addition of the bag-of-words features.

Comparing our results over the NEGATION subtask to *Negex*, it is evident that all results incorporating the *ERG *and/or bag-of-words features outperform this benchmark rule-based system, which is highly encouraging.

We were surprised by the effectiveness of the bag-of-words approach in comparison to our more informed techniques, particularly for NEGATION, where the simple bag-of-words baseline was superior to all other methods when combined with the UTurku Task 1 classifier. Nonetheless, the parsing techniques are clearly shown to have some utility (bearing in mind that there are still 7% of sentences which cannot be parsed under this setup thus will not be classified correctly from RMRS-derived features). However there is possibly room for improvement in the remaining 93% of sentences which we can parse - our results in Table 1 are still well below 93% recall.

We have not performed any analysis to verify whether the number of events per sentence differs between parseable and unparseable sentences. Longer sentences tend to be harder to parse, and may contain a larger number of sentences by virtue of their length, meaning that the true limit may be lower.

### Results over the test data

In the testing phase, we repurposed all of the development data as extra training data, and retrained using some of the promising combinations of RMRS sources and bag-of-words feature vectors. These results are presented in Table 2. Note that we are not able to evaluate over gold-standard Task 1 data, as it has not been released for the test data.

**Table 2:** Results over test set

Mod	RMRS from	Extra	R	P	F
SPEC	-	${\text{W}}_{+3}^{-3}$	6.97	23.73	10.77
SPEC	*ERG*	-	8.96	41.86	14.75
SPEC	fb(*ERG*, *RASP*)	-	12.44	52.08	**20.08**
SPEC	cb(*ERG*, *RASP*)	-	6.47	41.94	11.21
SPEC	*ERG*	${\text{W}}_{+3}^{-3}$	11.44	26.14	15.92
SPEC	fb(*ERG*, *RASP*)	${\text{W}}_{+3}^{-3}$	9.95	28.17	14.71 †
SPEC	cb(*ERG*, *RASP*)	${\text{W}}_{+3}^{-3}$	7.46	24.19	11.41

NEG	-	${\text{W}}_{+3}^{-3}$	19.55	30.94	23.96
NEG	-	*Negex*	17.83	12.73	14.85
NEG	*ERG*	-	11.82	35.62	17.75
NEG	fb(*ERG*, *RASP*)	-	13.64	34.09	19.48
NEG	cb(*ERG*, *RASP*)	-	12.73	33.33	18.42
NEG	*ERG*	${\text{W}}_{+3}^{-4}$	19.55	32.33	24.36
NEG	fb(*ERG*, *RASP*)	${\text{W}}_{+3}^{-4}$	19.55	32.58	24.43
NEG	cb(*ERG*, *RASP*)	${\text{W}}_{+3}^{-4}$	20.91	41.07	**27.71 **†

Results over the test data using the UTurku Task 1 system ("fb" = fallback strategy, where we use the first source if possible, otherwise the second; "cb" = use undifferentiated RMRSs from each source to create feature vectors). † denotes the feature set which performed best over the development set using gold Task 1 annotations.

The results here are not always what we would expect on the basis of the development results. The bag-of-words baseline continues to be an impressive performer for NEGATION, achieving an F-score of 24.0% with the UTurku data compared with 29.0% over the development data. However the combination of the aggregated RMRS approaches and the bag-of-words features outperformed bag-of-words.

The SPECULATION results show noticeably different behaviour from the development data. The primary difference seems to be that the bag-of-words baseline (at least for the context window that we selected) is of little use in comparison to the RMRS features. Encouragingly, the best result was obtained with a pure parser-based approach (fb(*ERG*,*RASP*)), and bag-of-words on its own was the poorest performer, with an F-score around half that of the parser-based method. This effect is even visible when combining the bag-of-words with the RMRS output, which resulted in a substantial decrease in F-score. Examining further, we can see that the bag-of-words recall is particularly low over SPECULATION, so it seems that the local contextual cues for SPECULATION which were learned from the training and development data are simply not present in the accessible events in the test data, while the longer distance syntactic dependencies are still clearly useful.

In terms of overall performance in comparison to the original submissions to the shared described in [1], these results are respectable. If we had been required to choose only one run for each of SPECULATION and NEGATION, the features would have been selected on the basis of the development set figures with gold Task 1 annotations (another option would be to use the best automatically created Task 1 annotations) - these figures are marked with '†' in Table 2. For SPECULATION, we would have submitted the fb(*ERG*,*RASP*), ${\text{W}}_{+3}^{-3}$ system to give an F-score of 14.71% giving results higher than the second-placed team, but well behind the score of ConcordU of 25.27% (the best performer over the test set would have been closer to the ConcordU performance, but this is not a fair comparison to make as it takes advantage of knowing scores over the test data). In the NEGATION subtask, using this technique would have selected the same parameters which gave the best test set performance, giving an F-score of 27.71% - higher than the top-ranked ConcordU score of 23.13%. Of course, in both cases these results rely on high-performing Task 1 systems from third parties which is important for Task 3 results, as we discuss below.

### Interaction between Task 1 and Task 3

**Figure 2:**
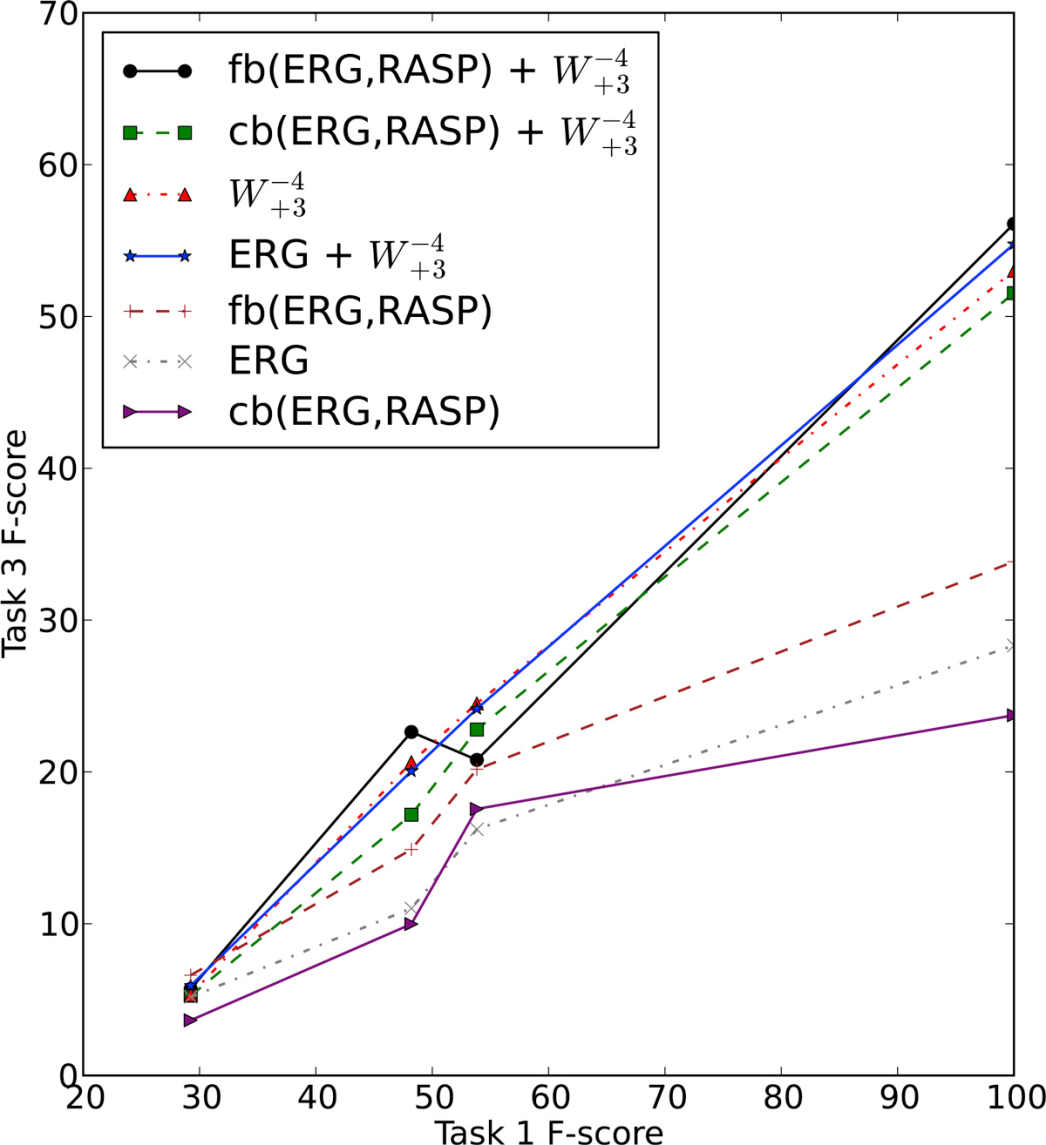
**Task 3 against Task 1 for SPECULATION**. Task 3 F-score against Task 1 F-score for SPECULATION, over the different combinations of Task 1 and Task 3 systems on the development set.

**Figure 3:**
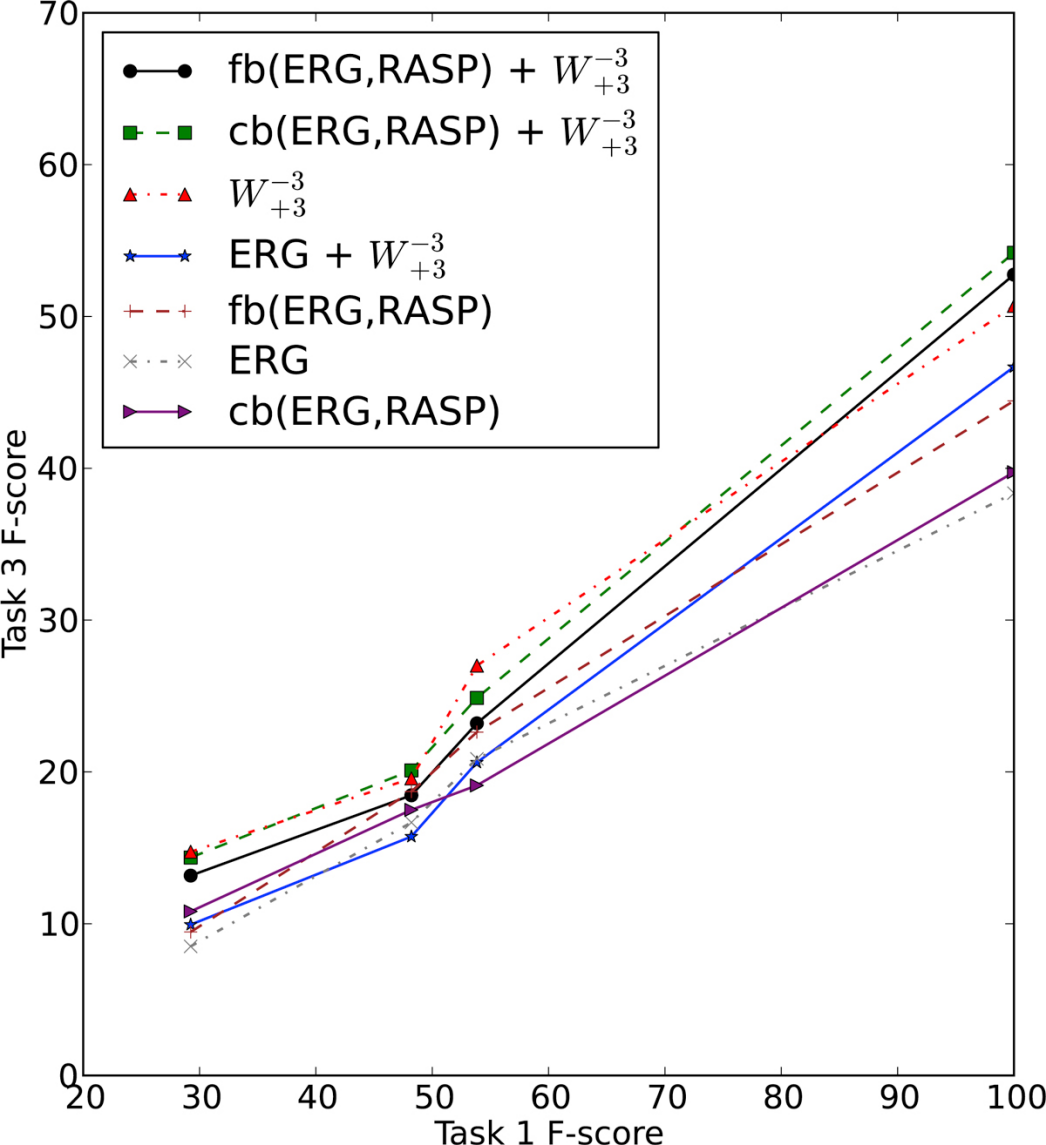
**Task 3 against Task 1 for NEGATION**. Task 3 F-score against Task 1 F-score for NEGATION, over the different combinations of Task 1 and Task 3 systems on the development set.

There is a clear interaction between Tasks 1 and 3 in our pipeline architecture, in that if there is an error in the Task 1 output for an event where there is SPECULATION or NEGATION, we have no way of correcting that mistake in our Task 3 classifier. What is less clear is the statistical nature of this interaction. To investigate this question, we plotted Task 3 performance relative to the performance of each of the three base Task 1 systems (UTurku, JULIELab and NICTA), over the various combinations of features. The results for NEGATION and SPECULATION are presented in Figure 2 and Figure 3, respectively. It is apparent from the two graphs that the correspondence is roughly linear, meaning that the relative gain in Task 3 F-score is roughly equivalent for every 1% gain in absolute F-score for Task 1. In the case of both SPECULATION and NEGATION, the slope of the various curves is relatively consistent at around 0.5, suggesting that it is possible to achieve a 1% increase in Task 3 F-score by boosting the Task 1 F-score by 2%. Of course, each of the curves in these graphs are based on only four data points, and there is inevitable noise in the output, but a rough linear trend is clearly demonstrated.

In the feature engineering stage, we primarily used the oracle data for Task 1 to maximise the amount of training data available. We felt that if we were to use our Task 1 classifications for events and trigger words, the effectively lower number of training instances would only hurt performance. However this possibly led to a bias towards features which were more useful for classifying events that were not successfully classified by the Task 1 system. The development set shows similar performance drops under these conditions in Table 1.

## Conclusions

We have presented a method for detecting event SPECULATION and NEGATION in bio-molecular literature, based on the BioNLP 2009 Shared Task data. We take a pipeline approach, in first detecting event trigger words and arguments (Task 1), then identifying occurrences of event modification based on this output (Task 3). Our method interprets modifier scope via the semantic output of the *ERG *and/or *RASP*, and presents this to a machine learner in the form of a linguistically-rich feature vector, which was optionally combined with bag-of-words features. We demonstrated that our parser-based approach was superior to a bag-of-words model for SPECULATION, achieving the best-published results over the SPECULATION subtask in the process. Surprisingly, for NEGATION, the simple bag-of-words approach was superior to all parser-based classifiers over the development data, but for the test data, the parsers achieved a higher F-score.

## List of abbreviations used

MRS: Minimal Recursion Semantics, a semantic formalism; RMRS: Robust Minimal Recursion Semantics, a formalism closely related to MRS; EP: Elementary Predicate, a unit of meaning in an MRS or RMRS; ERG: The English Resource Grammar, a handcrafted precision grammar of English; RASP: Robust Accurate Statistical Parser, a general purpose parser for English.

## Competing interests

The authors declare that they have no competing interests.

## Authors' contributions

AM implemented the Task 3 system, including parsing of sentences and feature engineering. DM handled the various Task 1 systems, and implemented the NICTA system for Task 1. TB co-ordinated the study and participated in its design. All authors read, contributed to and approved the final manuscript.
